# “We were afraid of the lion that has roared next to us”; community response to reactive focal mass drug administration for malaria in Eswatini (formerly Swaziland)

**DOI:** 10.1186/s12936-019-2877-9

**Published:** 2019-07-15

**Authors:** Kimberly A. Baltzell, Alysse Maglior, Khaya Bangu, Nontokozo Mngadi, Lisa M. Prach, Brooke Whittemore, Nyasatu Ntshalintshali, Manik Saini, Nomcebo Dlamini, Simon Kunene, Michelle S. Hsiang

**Affiliations:** 1 Dept of Family Health Care Nursing, UCSF, San Francisco, CA USA; 2 Institute for Global Health Sciences, UCSF, San Francisco, CA USA; 3 National Malaria Programme, Eswatini Ministry of Health, Manzini, Swaziland; 4 Clinton Health Access Initiative, Eswatini Office, Mbabane, Swaziland; 5 Malaria Elimination Initiative, Global Health Group, UCSF, San Francisco, CA USA; 6 Department of Pediatrics, University of Texas Southwestern Medical Center, Dallas, TX USA; 7 Department of Pediatrics, UCSF, San Francisco, CA USA

**Keywords:** Malaria elimination, Eswatini, Community acceptance, Malaria mass drug administration

## Abstract

**Background:**

Reactive focal mass drug administration (rfMDA), or presumptive treatment without malaria testing of household members and neighbours of a passively identified malaria case, is currently being explored as a possible malaria elimination strategy in low transmission settings. One of the primary factors determining the effectiveness of rfMDA on reducing or interrupting transmission is achieving high coverage of the target population with drug administration. This study aims to explore the acceptability of rfMDA and identify facilitators and barriers to its potential implementation, as well as the community’s general knowledge, attitudes and beliefs with regard to malaria elimination.

**Methods:**

A qualitative study was performed using focus group discussions (FGDs) among villagers that received rfMDA through the National Malaria Control Programme in the low transmission setting of Eswatini as part of a 2-year clinical trial. FGDs were audio-recorded, transcribed and translated into English. All transcripts were managed in Dedoose and underwent qualitative content analysis.

**Results:**

The majority of participants perceived their community to be at high risk of malaria. Witnessing others in their community suffer from malaria, proximity to Mozambique, various ecological factors, and the presence of mosquitoes contributed to this perception. The greatest motivator of participation in rfMDA was witnessing someone else suffer from malaria, since most participants had not personally experienced malaria themselves. Participants valued the education on rfMDA and on malaria in general, particularly when communicated by nurses and other health workers from the Ministry of Health. Participants were overwhelmingly motivated to participate in rfMDA in order to obtain protection from malaria. Most participants did not understand the concept of sub-clinical infection and, therefore, did not perceive the anti-malarial medication given in rfMDA to be a treatment medication.

**Conclusions:**

Perceived risk for malaria was a major driver of acceptability; therefore, future intervention campaigns could aim to better quantify risk to inform interventions and encourage uptake. There were misunderstandings about the asymptomatic reservoir of parasites in humans. Given that this phenomenon is the rationale for rfMDA, this misunderstanding could threaten the uptake of the intervention if it persists in the community. Using local authorities to deliver messaging, additional education on this concept with re-inforcement that risk of malaria is ongoing, even in the absence of frequent cases, may help to maximize and maintain acceptability.

## Background

Eswatini (formerly Swaziland) in southern Africa is one of several countries that have made significant progress towards malaria elimination [1, 2]. However, recent progress has stalled, indicating the potential need for new and more aggressive interventions [2, 3]. Currently, the main surveillance strategy used by the National Malaria Control Programme (NMCP) is reactive case detection (RACD), which is a form of active case detection (ACD) whereby surveillance officers test household members and neighbours of a passively identified index case, and treat those who test positive [4]. However, the effectiveness of RACD is limited by its reliance on rapid diagnostic tests (RDTs), which are inadequate for detecting the low density infections that currently comprise a high proportion of sub-clinical and asymptomatic cases [5–7]. Such low density infections may not be of major clinical relevance, but are sufficient to maintain the transmission of malaria in regions with very low malaria endemicity, such as in Eswatini [5, 8, 9]. Reactive focal mass drug administration (rfMDA), or presumptive treatment of household members and neighbors of a passively identified case without malaria testing, overcomes the limitations of the diagnostics used in RACD and may be effective for interrupting malaria transmission in low endemic settings [10–13]. Treatment with an anti-malarial agent that has a long half-life (e.g., dihydroartemisinin/piperaquine (DP) can also provide a prophylactic effect to those members of the community who are at highest risk of infection, even if they were not infected at the time of treatment [13].

rfMDA is being explored as a possible malaria elimination strategy in Eswatini, but there is limited information about its acceptability, which is critical for sustainability and effectiveness. One of the primary factors influencing the effectiveness of rfMDA to reduce or interrupt transmission is coverage, and it is estimated that at least 80% or even 90% coverage of the target population is necessary [14, 15]. Community participation, understanding and acceptance are essential in order to achieve this high level of coverage [8, 14, 15]. Acceptability of rfMDA could be a challenge in a low transmission setting where the perceived risks of treatment outweigh the low threat of malaria. Qualitative methods have been used in other settings to explore how local social circumstances and community engagement influence coverage of mass antimalarial drug administration [16–18]. This study aims to explore the acceptability of rfMDA, as well as the community’s general knowledge, attitudes and beliefs with regard to malaria elimination. The results of this acceptability study will be used to refine drug-based strategies for malaria elimination in Eswatini and other malaria-eliminating settings.

## Methods

### Study setting

A qualitative study was performed as part of a cluster randomized controlled trial (CRCT) to evaluate the effectiveness and feasibility of rfMDA compared to RACD in Eswatini (clinicaltrials.gov NCT02315690) [19]. Eswatini is a very low transmission setting with annual malaria incidence of 0.7 to 1.3/1000 population from 2012 to 2015 and parasite prevalence of 0.2% in 2010 [6, 20]. *Plasmodium falciparum* is the primary species and the principal vector is *Anopheles arabiensis*. Transmission is seasonal, mainly between October and May, and occurs in the eastern agricultural areas.

### Trial context

**Fig. 1:**
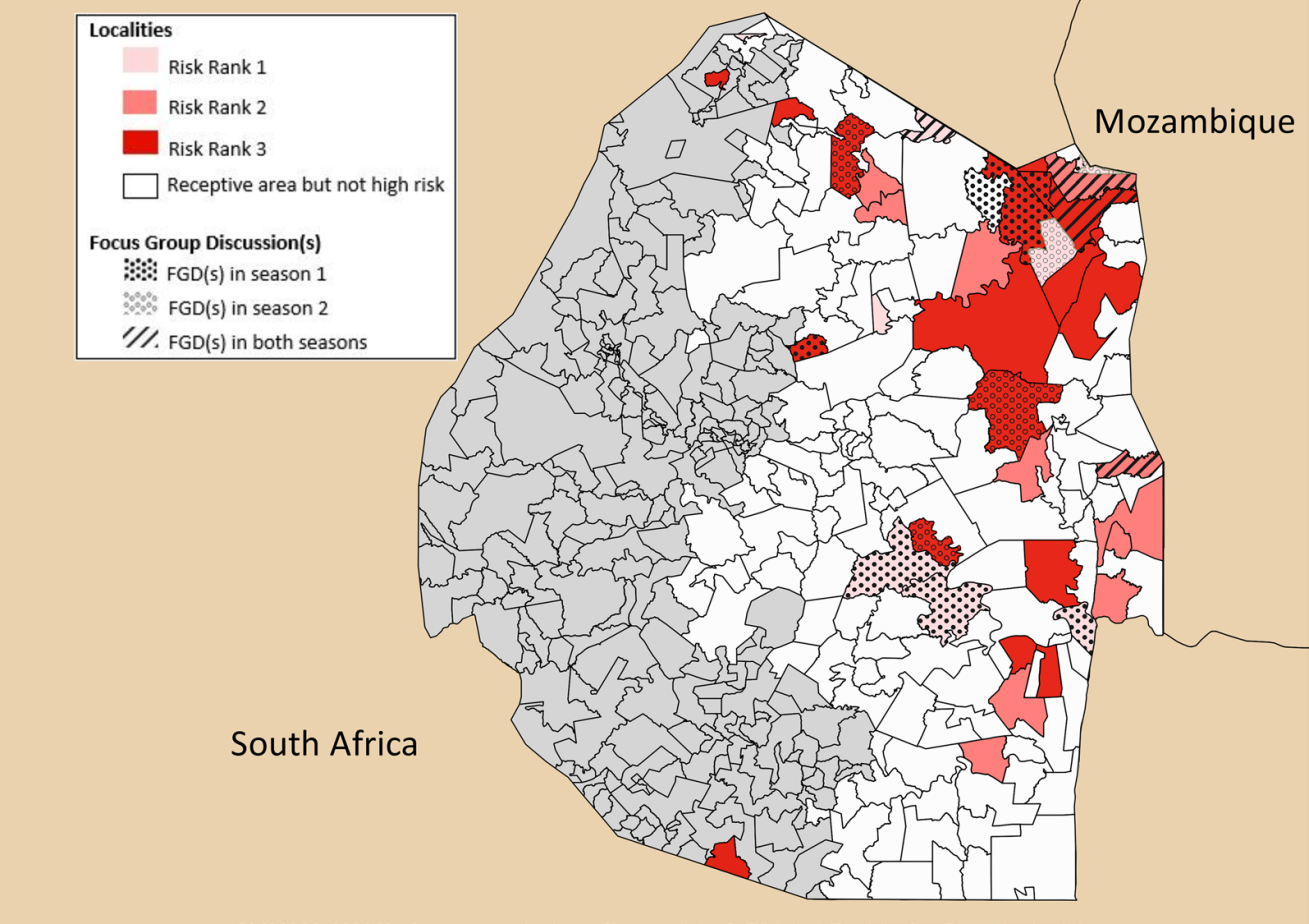
Study site in Eswatini

The primary aim of the CRCT was to determine if rfMDA is superior to RACD in reducing a primary outcome of incidence of passively detected malaria cases. Specifically, 77 of the highest risk localities in the eastern endemic areas of Eswatini were identified and randomized by risk rank and population size using a block stratified randomization method (Fig. 1). The trial took place from September 2015 to June 2017 and spanned two malaria transmission seasons: Season 1 (September 2015–June 2016) and Season 2 (July 2016–June 2017).

In the control arm, localities received RACD according to standard policy. The methods for case investigation [21] followed by RACD [4] have been previously described but, briefly, after a passively detected malaria case is reported through the national Immediate Disease Notification System, a surveillance team aims to visit the home of the index case within 48 h to conduct a case investigation. If the index case resides in the eastern endemic region, the team then aims to conduct RACD within a week. In RACD, all individuals residing within 500 m of the index case household are targeted for malaria testing by RDT (First Response *P. falciparum* HRP-2 Detection Test (Premier Medical Corp. Ltd) with RDT-positives referred to the nearest health facility for treatment with artemether-lumefantrine (Coartem, Novartis Pharmaceuticals Corp.) per national policy. In the rfDMA arm, instead of conducting RACD, the study team responded by screening all individuals within 200 m of the index case household for eligibility to receive the anti-malarial drug dihydroartemisinin–piperaquine (DP) (Eurartesim, Sigma-Tau IRF S.p.A), which is taken once daily for three consecutive days. All eligible and consenting individuals in the target area received the first dose of DP by directly observed therapy (DOT) and the remaining doses with self administration instructions were left with the participant or guardian. All individuals ineligible for DP were tested by RDT and referred to the nearest health facility for treatment if positive.

Prior to the trial and at least annually during the study, the NMCP conducted sensitization activities among health providers and the community. National and regional level meetings were held with physicians and nurses to review the protocol, educate regarding potential side effects associated with use of DP in largely asymptomatic individuals, and provide updates on study enrolment and safety. Informational meetings were held at tinkhundla-level meetings attended by chiefdom health representatives. Tinkhundlas are an administrative unit above chiefdoms. Each chiefdom has a health representative who is responsible for disseminating important information to the community. Similar meetings were held at the national level with rural health motivator trainers, or community health workers. The trainers then engaged rural health motivators. Prior to the implementation of study interventions, sensitisation was also conducted directly with study participants as part of the informed consent process.

### Study design

**Fig. 2:**
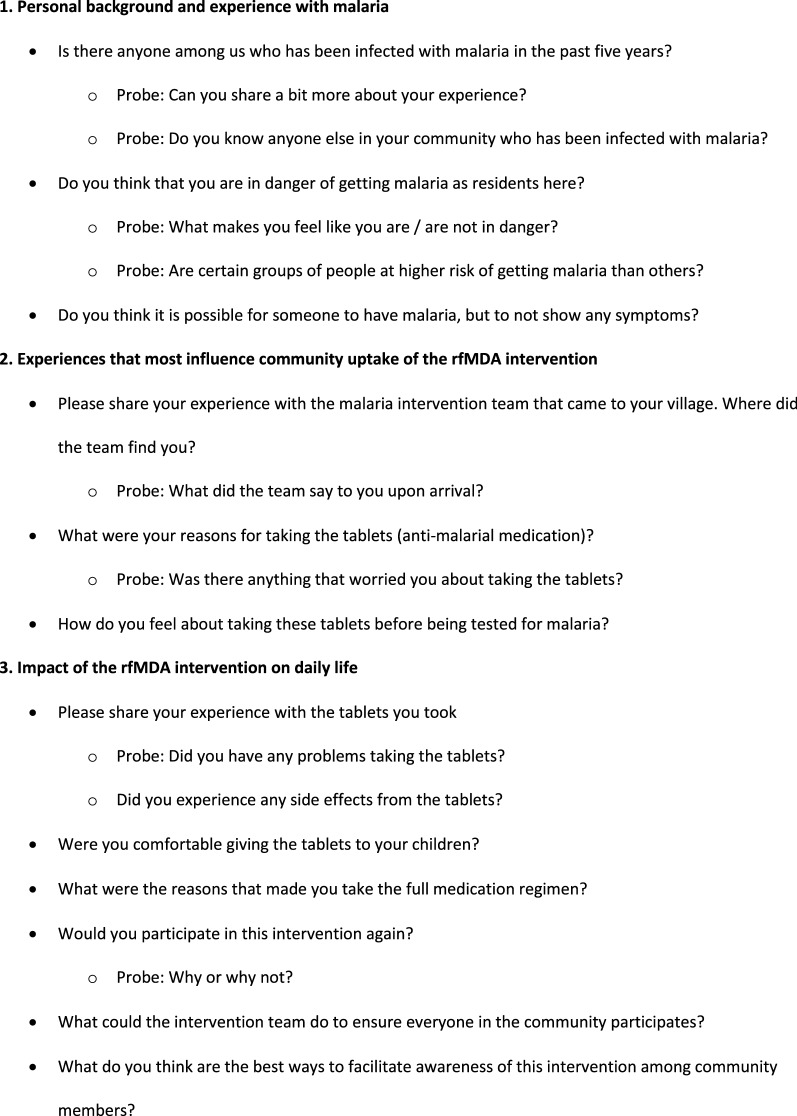
Focus group discussion sample questions

To assess the acceptability of rfMDA, a qualitative study was conducted using focus group discussions (FGDs) among community members that had recently received rfMDA. RACD communities were not studied as RACD has been a current standard of care intervention since 2009 and acceptability is high. FGD guides were developed to explore the following three areas of inquiry: (1) personal background and experience with malaria; (2) experiences that most influence community uptake of the rfMDA intervention; and, (3) impact of the rfMDA intervention on daily life. Representative sample questions are shown in Fig. 2. FGDs were conducted during two distinct periods, which corresponded with the transmission years in Eswatini: December 2015 to June 2016, referred to as Season 1, and July 2016 to June 2017, referred to as Season 2.

### Study population and sampling

**Fig. 3:**
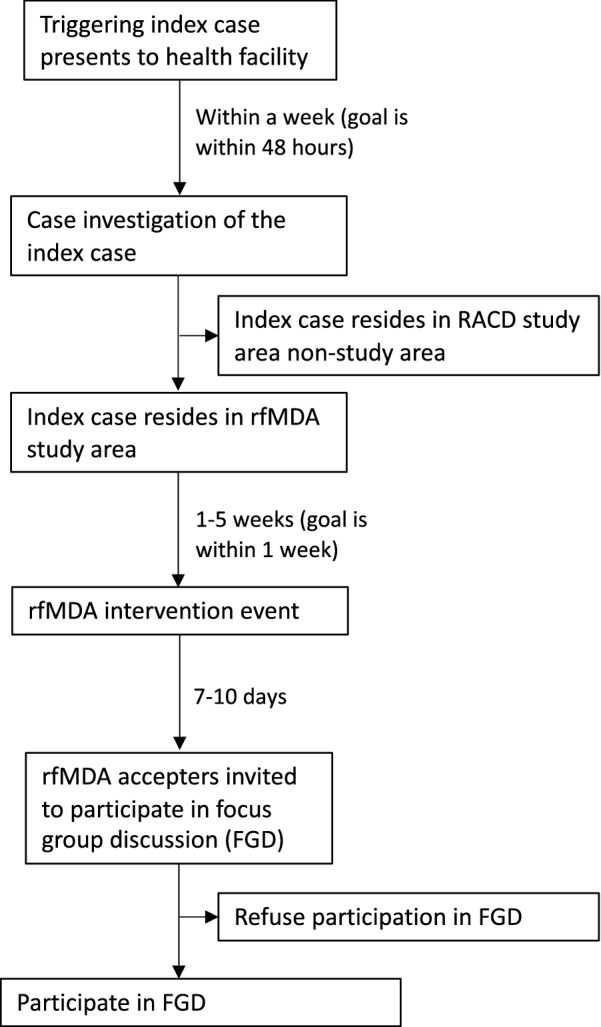
Flowchart of participant enrolment in qualitative study

The study population included people residing in localities that received rfMDA in 2015–2017 as part of the CRCT. At the time of trial enrolment, participating households were informed that the study team would return in seven to 10 days to conduct a pill count and potentially invite participants to join a FGD about their experience with the intervention. For FGDs, the study team sampled sites with the goal of conducting at least one FGD in each rfMDA locality per season. In selected sites, the study team returned seven to 10 days after rfMDA was implemented to recruit participants. Participants were eligible for inclusion if they were over 18 years of age, spoke the native siSwati language, participated in rfMDA, and provided informed consent at the time of the intervention. The study aimed to have relatively equal representation of both males and females. Five to 10 participants were recruited for each focus group based on their availability and willingness to provide written consent. A flow diagram of enrolment into the qualitative study is shown in Fig. 3. In addition, those who refused the intervention were offered a short anonymous survey to assist the study team in determining barriers to rfMDA.

### Data collection

After obtaining informed consent, semi-structured FGDs were conducted and moderated in the native siSwati language by local members of the rfMDA field surveillance team, who had been trained on topics such as malaria risk perception and experiences that may influence community members to accept rfMDA. All FGDs lasted approximately 1 h and were audio recorded. After the FGDs, experienced independent contractors transcribed the audio recordings in siSwati, and then translated the transcripts from siSwati to English. As FGDs took from participants’ meal preparation time, participants were provided lunch.

### Data analysis

The transcripts were thoroughly reviewed and imported into Dedoose (Dedoose Version 8.0.31, Los Angeles, CA, USA: SocioCultural Research Consultants, LLC) for management. Qualitative content analysis was performed, consisting of deductive and inductive elements. Two researchers independently conducted line-by-line coding of all data using a codebook that was partly pre-established from the initial research questions (deductive approach), but that was flexible enough to allow addition of codes as patterns emerged (inductive approach). Areas of disagreement were discussed until consensus was reached. The final codebook was agreed upon and applied to all transcripts. The content of the codes guided the development of the themes that are presented in the results.

## Results

Twenty-nine FGDs were conducted with 208 participants in 23 rfMDA intervention sites. Twenty-one FGDs were conducted during Season 1 (September 2015–June 2016) and eight FGDs were conducted during Season 2 (July 2016–June 2017). Each group consisted of 6–9 participants. Seven localities had FGDs in both Seasons 1 and 2, with unique participants in each focus group. While complete demographic information was not available for all FGDs given some participants were unsure of their age, all participants were over 18 years old, with an age range of 18–80 years (Table 1). Three main constructs formed the basis of the analysis: (1) risk perceptions, understanding and general experience with malaria and malaria interventions; (2) experiences that most influence community uptake of rfMDA; and, (3) impact of rfMDA on daily life. Of the 2008 eligible participants that were present and approached in the informed consent process, 1932 (96.2%) received DP and 76 (3.8%) refused DP. The refusal rate was 1.4% (11/776) and 5.3% (65/1232) in Seasons 1 and 2, respectively. Refuser data were only available from 9 of the 76 refusers. Some refusers cited more than one reason: dislike of medications (n = 2), personal experience with side effects of medicines (n = 2), child recently received medication at school for intestinal parasites and had side effects (n = 3), not notified in advance (n = 2), not available at this time (n = 1), and did not travel and therefore not at risk of malaria (n = 1).

**Table 1:** Focus group discussion (FGD) characteristics

	Season 1	Season 2	Total
Number of FGDs	21	8	29
Number respondents	156 (75.0%)	52 (25.0%)	208
Gender			
Female	86 (72.9%)	32 (27.1%)	118
Male	70 (77.8%)	20 (22.2%)	90
Age range (years)	18–78	18–80	

### Risk perceptions, understanding and general experience with malaria and malaria interventions

The majority of participants perceived their community to be at high risk of malaria, despite there being only a few participants who had experienced malaria themselves in the past. When comparing FGD responses between Seasons 1 and 2 in a sub-set of localities, perceived risk of malaria was higher in five of the seven localities in Season 2 compared to Season 1. Overall, there were four widespread reasons why participants perceived themselves to be at high risk of malaria. First, participants generally understood that mosquitoes transmit malaria, and they believed that they were at high risk due to the abundance of mosquitoes in their surroundings. One participant said,

*“The mosquitoes fly and have bitten, and made other people sick in the area recently. What is to stop it from biting me? I do not think anyone feels safe anymore.”* (Nyakatfo FGD participant)

Participants struggled to understand how some mosquitoes could be infectious while others are not. One participant said,

“*Can you clarify which mosquito exactly causes malaria? I do not think it’s the one that bites us everyday, because if it was we’d all be dead now”.* (Nyakatfo FGD participant)

Another said, *“I cannot see the dangerous mosquito.”* (Siphofaneni FGD participant). Although most participants associated malaria with mosquitoes, this understanding was not universal. In reference to a neighbouring case, a participant said,

*“I think we are at risk because we are not exactly sure of how he/she was infected. It could also be here, at home, we don’t know”.* (Luve FGD participant)

Another said,

*“We can’t think of anyone [who is at high risk of getting malaria] since we don’t know where it comes from”.* (Luve FGD participant)

Second, ecological factors contributed to perceived high risk of malaria. Specifically, rain and proximity to bodies of water, such as swamps, lakes and damp sugar cane fields, were perceived to be risk factors by attracting mosquitoes and promoting breeding. One participant said,

*“We think we are in danger as we live next to still water and ponds…I say this because stagnant water causes the mosquitos to breed and the water that drips from the taps you find that it settles and the mosquito can breed there and come back to sting us”.* (Emalibeni FGD participant)

Puddles of muddy water on a homestead or empty fish tins that have collected water were also regarded as mosquito breeding grounds. There was a general sentiment that these conditions were undesirable and associated with people maintaining unclean homes. There was also general sentiment that vector control interventions, such as indoor residual spraying (IRS) and insecticide-treated bed nets were necessary, with many participants expressing interest to receive bed nets during interviews.

Third, witnessing others in their community suffer from malaria made the threat of malaria seem imminent:

*“We are not safe because we are close to the sick person. We don’t know how they were infected except that we try to be on the lookout for the malaria symptoms so that we can be protected.”* (Maphobeni FGD participant)

All focus groups had participants who knew someone in the community who had malaria in the past, and seven of the focus groups had participants who explicitly reported that they believed they are at high risk of malaria because of these neighbouring cases.

Fourth, participants felt at risk in their communities *“because we are next to the Mozambican border*”. *(Mahlahlane FGD participant).* Another participant said,

*“I say [we’re at risk] because we are closer to Mozambique and even here in [Community Name] there is the malaria causing mosquito.”* (Mafucula FGD participant)

Mozambique was generally perceived to have higher risk of malaria compared to participants’ own communities, so frequent travel to Mozambique was considered a high-risk activity *“because they [Mozambique] are the ones who have a big problem with malaria yet they are our neighbours.”*(Lomashasha FGD participant).

### Experiences that most influence community uptake of rfMDA

#### Motivations to participate in rfMDA

Participants generally felt positive about rfMDA. Perhaps the greatest motivator of participation in rfMDA was witnessing someone else suffer from malaria. Since most of the participants had not personally experienced malaria, knowing someone else with malaria motivated community members to participate in rfMDA. One participant said,

*“I have seen people die in my presence…they died as result of malaria. That is why I quickly accepted.”* (Khomba FGD participant)

Participants were more highly motivated to participate if the index case lived in very close proximity, such as a household member or a child. Knowing a neighbour slightly farther away who had malaria was also motivating, but some participants believed the risk of infection belongs to only those in the same household.

I: *Let’s assume that they are your neighbours. As she has explained that her partner has once had malaria, my question is that do you consider yourselves at risk of getting malaria?*

P: *I wouldn’t consider myself to be at risk; maybe I can be if I stay with this person in the same homestead.*

I: *When it’s the neighbour that is infected you don’t have a problem of getting malaria?*

P: *I think we are far enough, maybe.* (Maphobeni FGD participant)

There were a few aspects of the rfMDA implementation that stood out to the participants as appealing. First, participants appreciated the presence of nurses on the implementation team who were administering the anti-malarial medication. For example, one participant said,

*“It’s important to listen to the nurse who cares about your life and she is educated on it…Otherwise how can you answer her when she comes and you have some [medication] unused? Because if you have followed instructions fully, you can have the confidence to inform her it didn’t work.”* (Mafucula FGD participant)

Community members have widespread respect for nurses, which reportedly increased their confidence in taking the full medication regimen. It was also reported that simply seeing the nurses in uniform increased participants’ confidence in the implementation team.

Second, ample time spent by the implementation team educating the communities on malaria and on the rationale behind rfMDA before rfMDA implementation helped to build rapport with community members and increased their willingness to participate in rfMDA. This pre-implementation educational messaging facilitated a deeper trust of the implementation team and a greater understanding of the importance of participation among community members.

P*: They [the implementation team] said that once I have taken the medication, even if the mosquito may bite me, it would not harm me. Also, if I take the medication, I must finish the whole course.*

I*: If I may ask what or who convinced you to eventually take the pills?*

P*: It’s the way it was fully explained to us…that once you have taken the pill, you achieve this and that, hence our acceptance.”* (Engulubeni FGD participant)

Community members also reported increased confidence in the implementation team because the team identified themselves as *NMCP brought them, I trust them, they take care of us. They give us nets and they spray our houses. Hence, I wasn’t afraid, because it is for our own good.”* (Nyakatfo FGD participant).

There was a general sentiment of trust and acceptability towards government and government programmes. Participants felt more comfortable when the entire implementation team was wearing the same uniform/logo shirt. The uniformed team members and the presence of the NMCP logo increased credibility and acceptability of the team and, therefore, of rfMDA.

Another widespread motivation for family-wide participation in rfMDA was participants’ desire to keep their children healthy and prevent them from getting malaria. One participant said,

*“I stay alone with two children. If I refuse to protect myself from mosquitoes, the children could be sick and that would affect their school attendance.”* (Khomba FGD participant)

Participants expressed concern that rfMDA implementation teams visited the communities while children were still in school, limiting the ability to protect their children. There were two participants, however, who stated concern for children taking medication because of potential adverse side effects due to the child not being as able to resist reactions as an adult might.

### Understanding of rfMDA rationale

There were many statements indicating confusion around the idea of a parasite reservoir. Many participants felt symptoms needed to be present to be infected or infectious. Accordingly, many participants did not understand that the mechanism of the drug used in rfMDA (DP) is to treat sub-clinical infections with the added bonus of providing short-term protection. Most participants believed that the medication they were taking was strictly for prevention, and that symptomatic individuals would take a different medication for treatment.

*“I think [treatment before testing] is not good because it might happen that you have the virus and they will give you prevention tablets instead of treatment.”* (Emalibeni FGD participant)

This misunderstanding led to focus group participants insisting on receiving testing before treatment, but in the same breath quickly agreeing to take ‘prevention tablets’.

“P: *It is better that you run the tests before giving us the tablets…It is better I undergo check*-*ups first.*

I: *You want to get sick first before you get prevention?*

P: *It is better that you give me the tablet…even at first I agreed with prevention because I want to be safe.”* (Mahlahlane FGD participant)

There were a few participants, however, who did understand the implications of asymptomatic malaria. Messaging around the concept of a parasite reservoir was clear to FGD participants from one locality in particular. One participant from this community explained,

*“It can happen as you can find that it is still in its early stages. It cannot show symptoms after stinging you the very same day, it moves along the body for some time before the symptoms can be revealed. Then that is when it can show this stage.”* (Thabankulu FGD participant)

However, it was unclear if participants felt medication given during rfMDA would target early stages of infection. While confusion persisted around treatment *versus* prevention pills in Season 2, participants in two of the seven localities that received FGDs both seasons demonstrated a clearer understanding of infection reservoir in Season 2 FGDs.

### Impact of rfMDA on daily life

#### Tolerability of the rfMDA drug regimen

The anti-malarial drug was generally well tolerated. Consistent with the pill count findings (99.3% adherence, n = 1114, unpublished), mostly all participants reported completing the full medication regimen, even when side effects were experienced. The reported side effects were mild and included weakness, dizziness, nausea/vomiting, stomach ache, headache, and fever. The majority of participants said they would take the medication again because they know the benefit of malaria prevention outweighed the cost of side effects. One participant, when asked why he and others completed the medication despite side effects, explained,

*“We were afraid of the lion that has roared next to us. You were not going to leave [the antimalarial medication] without finishing it as [the mosquito/malaria] has roared.”* (Lomahasha Etigodzini FGD participant)

Only one participant explicitly said they would not take the medication again due to the experienced side effects.

#### Community engagement

When asked what malaria programmes could do to help the community, many participants requested bed nets. However, other participants thought that ‘prevention tablets’ (referring to the anti-malarial medication in rfMDA) were a better malaria prevention tool than bed nets because the tablet can provide protection at all hours of the day:

*“You can aid by giving us the tablets as they are of good health because some other time when you are outside seated at home a mosquito can sting you during the day yet you only need the net to prevent you when you are sleeping in your bed so it is risky sometimes yet with the tablets you are always protected.”* (Embasheni FGD participant)

Regarding community sensitization activities, community members expressed desire to have more interface with the NMCP and more time to understand rfMDA before a team arrives to conduct the intervention. More educational materials and messaging on rfMDA and malaria in general were desired from participants in all FGDs. Participants suggested messages around rfMDA should emphasize the importance of how rfMDA could provide protection against malaria for the entire community if each person participates.

*“Visit (Community Name) more often and pin notices all over the community inviting people to attend malaria educational talks. That should help. You will be amazed with the number of people who will attend. Even a person who would have refused will be motivated, they won’t want to be the odd one out.”* (Nyakatfo FGD participant)

Participants across 15 different FGDs suggested that malaria related health promotion activities similar to HIV educational messaging would be effective. Specifically, there were 18 quotes about health talks in the communities, 11 quotes about radio and TV segments, and three quotes about advertisements or posters at bus stops and shops. A few participants also mentioned incorporating the rural health motivators and incorporating malaria education into schools. For example, one participant said,

*“It can also be gainful if you can find some days and teach learners in the various schools, early before they start their school lessons. There you can teach them on all your malaria related issues.”* (Engulubeni FGD participant)

In addition to educational messaging, a consistent NMCP presence in the communities would help to increase motivation and engagement with rfMDA. This finding was consistent when comparing Season 1 and Season 2 findings at the same sites. One participant noted that changing cultural practices takes time:

*“You can also introduce some support groups which can teach even once a month. This would help reinforce the information to the people because Africans take time to accept changes. It takes time for them to even unlearn some deep cultural or traditional practices.”* (Nkalashane FGD participant)

Lastly, participants mentioned that while the majority of community members would likely continue to accept rfMDA if they were continually engaged with the strategies suggested above, there is a small group of ‘others’ in the community that think differently and would be a harder audience to convince. One participant provided reasons for lack of acceptance,

*“It depends on the person, you cannot say you have a way of making him agree with what you are saying. You can come with your tablets and that person denies them, you come with your net and they refuse to use it. So I think it depends on the person, because others are afraid of injection and they also say the tablets are making them sick. You see such a thing.”* (Siphofaneni FGD participant)

Lack of acceptability towards other NMCP interventions, such as IRS, a core malaria intervention in Eswatini, were also expressed:

*“Some people lock their houses and leave as soon they see NMP officials spraying in the neighborhood. You find that in some cases they lock their houses, go to the forest to collect firewood and get bitten by the mosquitoes. This thing is a lot of work. When they come to spray, furniture has to be moved to the center of the house…[laughter] you have to prepare yourself. I agree with the young lady, people need to be educated and then they make their own choices. People are so thickheaded; they tell you that if they fall sick, they will go to (name of health centre). They irritate the nurses and complain that nurses aren’t giving them the necessary attention, yet they forget that when the NMP came for spraying, they disappeared. You hear them say “I wasn’t at home when the spraying was done”, forgetting that it was them that locked their houses and went to collect firewood.”* (Nyakatfo FGD participant)

FGD participants suggested additional messaging to maximize community participation in the different NMP interventions.

## Discussion

This qualitative study aimed to assess the community acceptability of rfMDA, a new malaria elimination strategy, and to glean more information on the general knowledge and beliefs of malaria transmission and elimination of the study area communities in Eswatini. Overall, this study found that community members were very receptive to taking anti-malarial medication without testing. Key drivers of acceptability were: (1) perceiving oneself or family member to be at risk for malaria; (2) a nuanced understanding of the concept of a parasite reservoir; and, (3) receiving educational messaging about rfMDA, specifically about the benefit of community-wide participation, from credible members of the community. Findings from these three major themes illuminate areas that may need to be addressed or reinforced if rfMDA is adopted and implemented as part of Eswatini’s malaria elimination programme. These findings also have implications for other settings that are implementing drug-based strategies for largely asymptomatic populations. Further, the findings align with the existing literature; it is clear that an understanding of malaria transmission and educational messaging before an intervention are key for acceptability of other mass drug administration programmes, including ones for malaria [16, 22, 23].

Motivation to participate in rfMDA is important to address in areas of declining malaria transmission because asymptomatic individuals may not feel that taking medication is necessary. Theories of behaviour change suggest using specific ‘drivers’ to motivate community members to accept protective measures [24, 25]. Recent research shows ‘affective’ risk perception (e.g., worry, anxiety or fear) is the strongest predictor of protection motivation [26] compared to reason-based judgement or feelings of vulnerability. Focusing on community education regarding the impact of continuing transmission on their community and the ubiquitous nature of sub-clinical infections rather than individual ‘carriers’ may be effective in improving the uptake of interventions. Additionally, the presence of uniformed nurses at the rfMDA intervention increased the confidence and sense of urgency of community members to participate in rfMDA due to the widespread respect for nurses. The current standard of care, RACD, deploys surveillance agents to perform the testing and referral to a health facility for treatment, if positive. Even if RACD were continued, the programme could consider incorporating nurses to help ensure high coverage.

An integrated approach to delivering interventions for malaria includes a focus on vector control, diagnosis and treatment, and chemoprevention [27]. In this study, participants expressed interest in interventions that target the mosquito vector as well as humans, with many participants requesting bed nets at the time of interviews. Bed nets are no longer distributed by the government due to low usage in the past, but the IRS programme is active [28]. Whether bed nets should be used in addition to IRS is beyond the scope of this study, but it is important to note that community members were interested in a strategy that addresses both humans and the mosquito. An rfMDA trial in Namibia is currently evaluating the effectiveness of rfMDA and reactive focal IRS, which could be considered if proven effective and acceptable to the community [13].

Messaging about malaria in general and about the rationale behind rfMDA was key for community members to understand how and why rfMDA may be important for elimination. Participants repeatedly said that emphasis on the importance of protection from malaria in educational messaging is critical for rfMDA uptake. Otherwise, community members may eventually refuse to participate if their perception of risk of malaria infection decreases. Lessons from Southeast Asia, where community members were also trained as volunteers and formed part of the study in community engagement activities, may inform useful strategies in Eswatini [17, 29, 30]. Similarly, polio eradication campaigns in India successfully used networks of social mobilizers (usually females from the community) to ensure uptake of vaccinations despite communities having no recent exposure to polio [31]. Some participants echoed this, suggesting rural health motivators to help with messaging. The community also requested repeated opportunities to receive information through varied venues including in lectures and the media. Although sensitization activities were implemented pre-trial and at the time of rfMDA, it did not seem to have the anticipated reach.

Through both Seasons, there was some confusion and inconsistency regarding understanding around human reservoirs and medication for treatment versus prevention. Participants’ comments revealed potential variable quality and/or effectiveness of pre-implementation educational messaging provided by the intervention teams on this topic. Community members understood and were pleased that the pills administered in rfMDA helped to prevent malaria. However, due to poor understanding of sub-clinical malaria, which has also been described in other settings [32], participants seemed to be unaware that rfMDA also served to presumptively treat any existing reservoir. That this concept goes largely against past messages regarding the need to only treat laboratory-confirmed malaria in health clincs likely contributed to the confusion. That the intervention was conducted with DP, and not artemether-lumefantrine, which is normally used for treatment, may have inadvertently led to a focus on prophylaxis over treatment. Encouraging intervention teams to emphasise the treatment benefit of anti-malarials in addition to the prophylactic benefit may help facilitate adherence to the medication as well as community level coverage (e.g., community members may encourage other community members to receive treatment due to potential community level benefit of transmission reduction). Further, outside of studies of intermittent preventative treatment [33] and one large-scale MDA study [34], the prophylactic effect of DP in one-off use, as in this study, has not been well studied. Also, overemphasis on the prophylactic effect may lead to a false sense of security, leading to decreased uptake of other preventative interventions and decreased health seeking. As such, increased clarity and consistent quality [35] regarding the delivery of messaging regarding rfMDA benefits is necessary for community understanding and engagement.

The side effects reported in this study were similar to other reports of anti-malarial medication side effects, including fatigue, headache, dizziness, nausea, and vomiting after taking the anti-malarial medication [36]. Despite these reports, most participants’ fear of getting malaria seemed to override the desire to avoid side effects and motivated participants to complete the medication regimen. Participants understood that by stopping the medication they would have a weaker defence against malaria than before, and they knew the side effects were only temporary.

Limitations of the study include the possibility of recall bias. However, FGDs were conducted within 10 days of an intervention. Additionally, FGDs were primarily conducted during the day and therefore findings may not fully reflect all members of the community, especially those who work during the day. It was not possible to conduct individual interviews due to the difficulty of accessing the sites and limited time during pill count visits. This may have narrowed the ability to get a more nuanced understanding of acceptance. Further, there were more women than men participants, perhaps reflecting the work demographic in the study localities. It is possible that women were more motivated to participate given their concern for their children’s health and primary role as caregiver. There is also potential for selection bias as the study only included community members who agreed to participate in rfMDA. However, refusal rates for rfMDA were low suggesting selection bias was likely limited. The provision of a meal was unlikely to have influenced response rates or responses because it was only offered after the FGD was completed.

There were several strengths of this study. First, the high volume of FGDs conducted with rfMDA participants enabled this study to reach saturation of themes and to confidently identify the findings presented here. Second, FGDs were conducted over a 2-year period, enabling the study to observe changes in acceptability over time. Another strength is that feedback to the intervention team was provided in real-time to help inform implementation. For example, the increased perception of risk in Season 2 versus Season 1 prompted clearer messaging about the rfMDA rationale and malaria risk to the community during rfMDA administration.

## Conclusions

This qualitative study of a new drug-based intervention directed at largely asymptomatic populations in Eswatini found several areas that may need to be addressed or reinforced should rfMDA be implemented on a larger scale. Given that perceived risk for malaria was a major driver of acceptability, future intervention campaigns can aim to better quantify risk (including risk of resurgence when transmission is very low) to inform interventions and encourage uptake. Also, there were misunderstandings about the asymptomatic reservoir of parasites in humans. Given that this phenomenon is the rationale for rfMDA, this misunderstanding could threaten the uptake of the intervention if it persists in the community. Additional education on this concept with reinforcement that risk of malaria is ongoing, even in the absence of frequent cases, may help to improve and maintain acceptability. Finally, delivery of educational messaging and interventions from trusted individuals, along with stronger integration with other malaria interventions, will also be key to community engagement.

## Data Availability

The dataset supporting the conclusions of this article can be requested from the Eswatini Ministry of Health.

## Abbreviations

rfMDA: reactive focal mass drug administration
FGD: focus group discussion
NMCP: National Malaria Control Programme
RACD: reactive case detection
ACD: active case detection
RDT: rapid diagnostic tests
DP: dihydroartemisinin/piperaquine
CRCT: cluster randomized controlled trial
IRS: indoor-residual spraying
